# Supplementary material for: Emergence of bacterial glass
Source: PNAS Nexus. 2024 Jun 14;3(7):pgae238. doi: 10.1093/pnasnexus/pgae238 (PMC11238424; doi:10.1093/pnasnexus/pgae238)
Supplement: pgae238_Supplementary_Data [file pgae238_supplementary_data.zip › SupplRevised.pdf]

Supplementary material for "Emergence of bacterial glass"

Hisay Lama,[1] Masahiro J. Yamamoto,[2,1] Yujiro Furuta,[3,4] Takuro Shimaya,[1,4] and Kazumasa A. Takeuchi[1,4,*]

[1]*Department of Physics, The University of Tokyo, Tokyo, Japan*
[2]*National Metrology Institute of Japan, National Institute of Advanced Industrial Science and Technology, Tsukuba, Japan*
[3]*Department of Physics, Tokyo Metropolitan University, Tokyo, Japan*
[4]*Department of Physics, Tokyo Institute of Technology, Tokyo, Japan*

(Dated: July 4, 2024)

Mode-coupling theory exponent γ

Our bacterial glass transitions show a rapid increase of relaxation times, τ_Q and τ_θ for translational and orientational degrees of freedom, respectively, as the area fraction ϕ is increased (Fig. 2c symbols). The observed dependence on ϕ is consistent with the power-law divergence that mode-coupling theories (MCT) predict [1–3] (Fig. 2c dashed lines),

$$\tau_Q \sim (\phi_{\mathrm{c}}^Q - \phi)^{-\gamma_Q}, \qquad \tau_\theta \sim (\phi_{\mathrm{c}}^\theta - \phi)^{-\gamma_\theta}, \tag{S1}$$

as well as with the Vogel-Fulcher-Tamman (VFT) law [1, 2, 4] (Fig. S6).

Here we focus on the exponent γ of the MCT power laws, Eq. (S1). Our fitting gives $\gamma_Q = 1.6(3)$ and $\gamma_\theta = 1.5(13)$ (see main text). In contrast, for thermal systems, it is actually known that MCT generally gives $\gamma \geq \gamma_0 \equiv 1.76\ldots$ [1]. More precisely, for spherical particle systems near equilibrium, one may indeed prove $\gamma \geq \gamma_0$ [1]. The situation is more involved for the aspherical case, where the same inequality has not been proven from first principles, but by using a diagonalization approximation of the MCT memory kernel, one can still show $\gamma \geq \gamma_0$ [5]. This inequality has also been confirmed by simulations of different aspherical particle systems (e.g., [6, 7]), without any exception so far, to our knowledge. Therefore, it is reasonable to consider that $\gamma \geq \gamma_0$ generally holds for systems near equilibrium. Our estimate $\gamma_Q = 1.6(3)$ seems to violate this bound, thereby indicating the non-equilibrium nature of our bacterial system. This observation raises some interesting questions that may deserve further investigation, such as clarifying conditions to violate the inequality $\gamma \geq \gamma_0$, how generally this violation takes place in non-equilibrium or active systems, etc.

* kat@kaztake.org

[1] W. Götze, *Complex Dynamics of Glass-Forming Liquids: A Mode-Coupling Theory*, International series of monographs on physics (Oxford Univ. Press, New York, 2009).
[2] L. Berthier and G. Biroli, Rev. Mod. Phys. **83**, 587 (2011).
[3] D. R. Reichman and P. Charbonneau, J. Stat. Mech. **2005**, P05013 (2005).
[4] G. L. Hunter and E. R. Weeks, Rep. Prog. Phys. **75**, 066501 (2012).
[5] R. Schilling and T. Scheidsteger, Phys. Rev. E **56**, 2932 (1997).
[6] A. J. Moreno, S.-H. Chong, W. Kob, and F. Sciortino, J. Chem. Phys. **123**, 204505 (2005).
[7] P. Pfleiderer, K. Milinkovic, and T. Schilling, Europhys. Lett. **84**, 16003 (2008).

SUPPLEMENTARY FIGURES

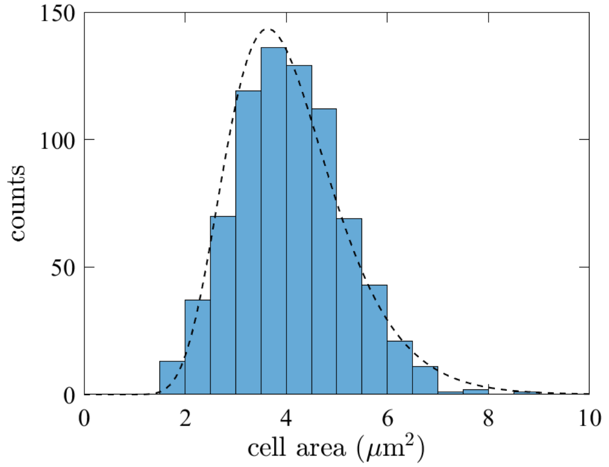

FIG. S1. Histogram of cell areas. The cell areas were evaluated for $\phi = 0.784(7)$, from the first three frames where the cell segmentation was carried out. The dashed line shows the fitted log-normal distribution, whose probability density is given by $\frac{1}{\sqrt{2\pi}\sigma x} \exp\left[-\frac{(\log x - \mu)^2}{2\sigma^2}\right]$ with $\mu = 1.37(2)$ and $\sigma = 0.281(15)$ (the value of x is in the unit of μm^2). Here the uncertainty corresponds to the 95% confidence interval from the fit. The mean cell area is 4.08 μm and the polydispersity index is 1.07. Note that a few filamentous cells and cells at the boundary of the region of interest were excluded from this histogram.

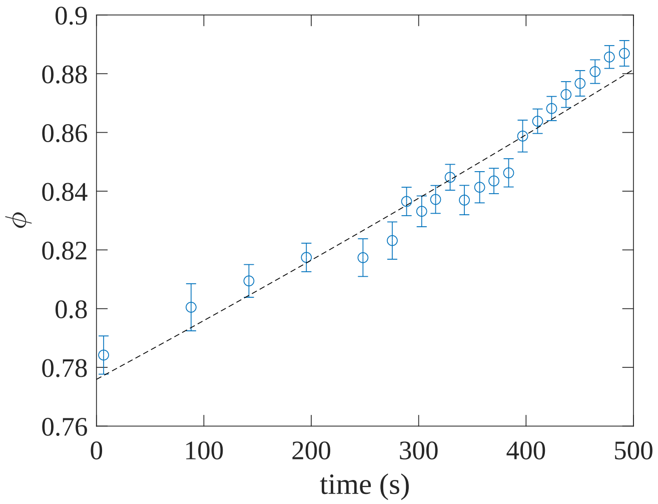

FIG. S2. Growth of the area fraction ϕ. The area fraction ϕ was evaluated for each group of 500 images recorded over $\approx$ 13 s. The time point at the center is used in this plot. The dashed line shows a fit to the exponential growth curve, $\phi(t) \propto 2^{t/T}$, which estimated the doubling time at $T = 45(5)$ min. Here the uncertainty corresponds to the 95% confidence interval from the fit. Note that the origin of time is set to be the moment at which we started the acquisition of the first set of images, which was already roughly five hours after the start of the experiment.

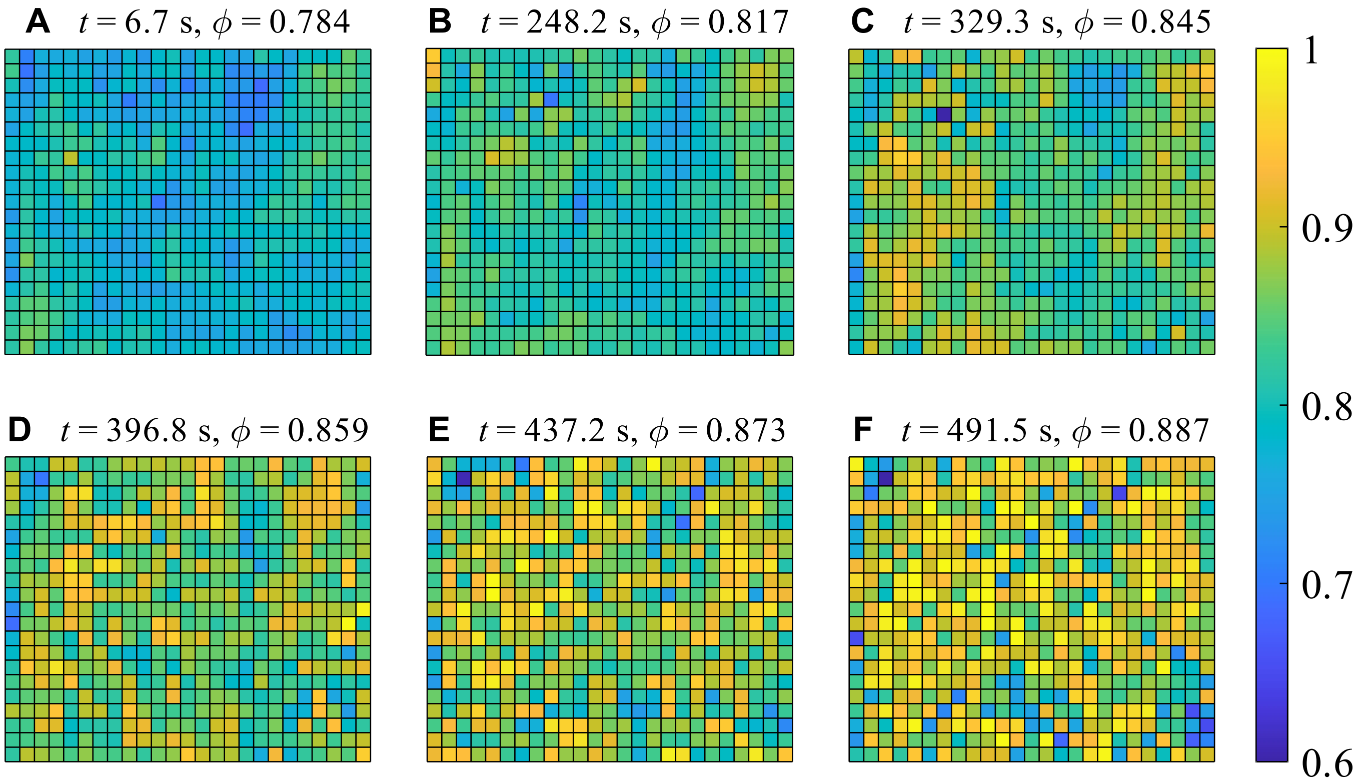

FIG. S3. Spatial dependence of the area fraction ϕ. The area fraction ϕ was evaluated for each group of 500 images recorded over ≈ 13 s, locally in a mesh composed of regions of 10×10 pixels ($1.724\ \mu$m $\times$ $1.724\ \mu$m). Above each panel, the time at the center of each time interval and the mean value of ϕ (averaged over the time interval and the region of interest) are displayed. Note that the origin of time is set to be the moment at which we started the acquisition of the first set of images, which was already roughly five hours after the start of the experiment. These indicate that uniform growth was indeed realized in our device.

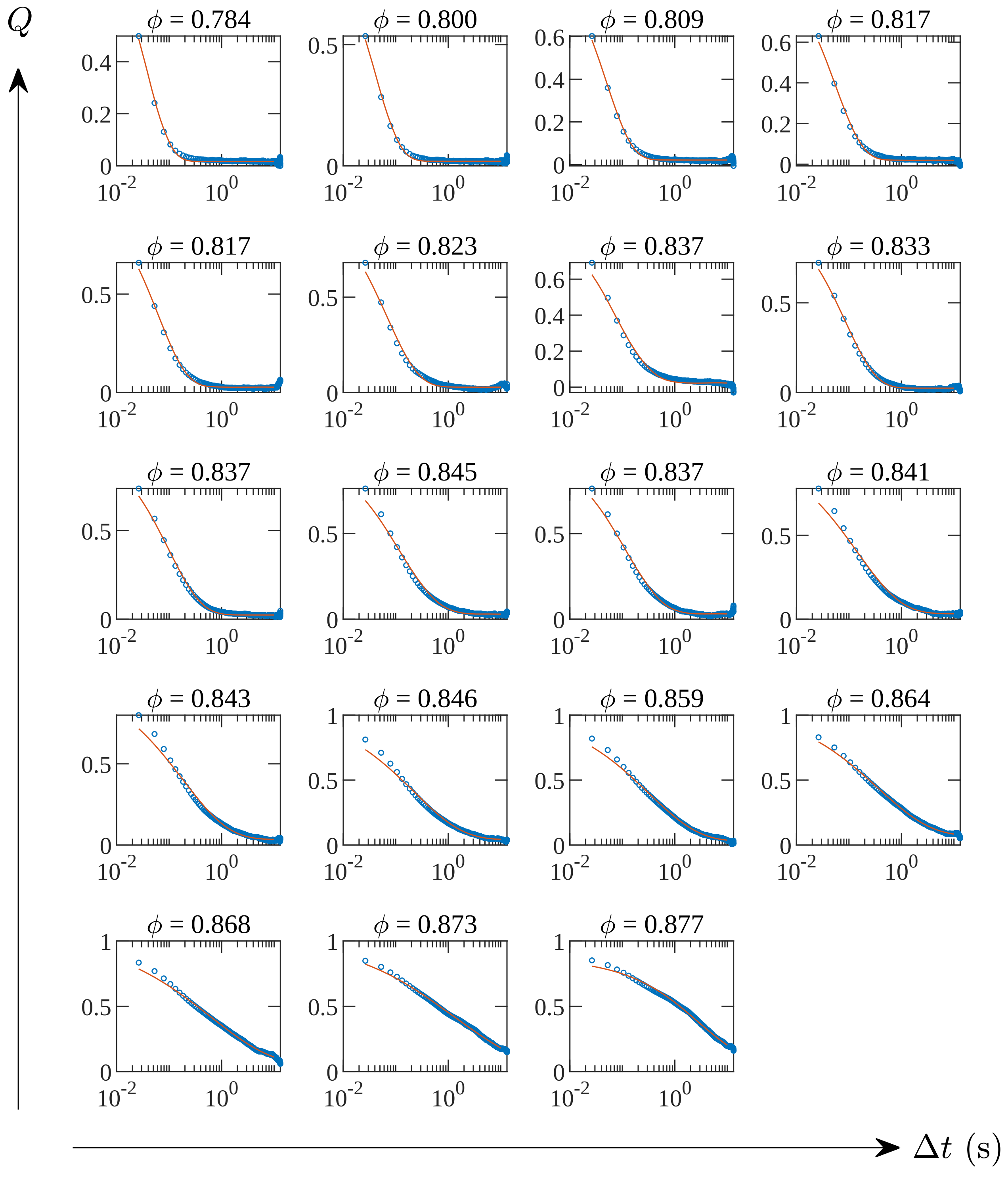

FIG. S4. Results of fitting of the overlap function $Q(\Delta t)$ by a stretched exponential function. Each data was fitted by a stretched exponential function plus an offset, $Q(\Delta t) = f_Q e^{-(\Delta t/\tau_Q)^{\beta_Q}} + a_Q$. See Fig. S5 for the obtained values of β_Q.

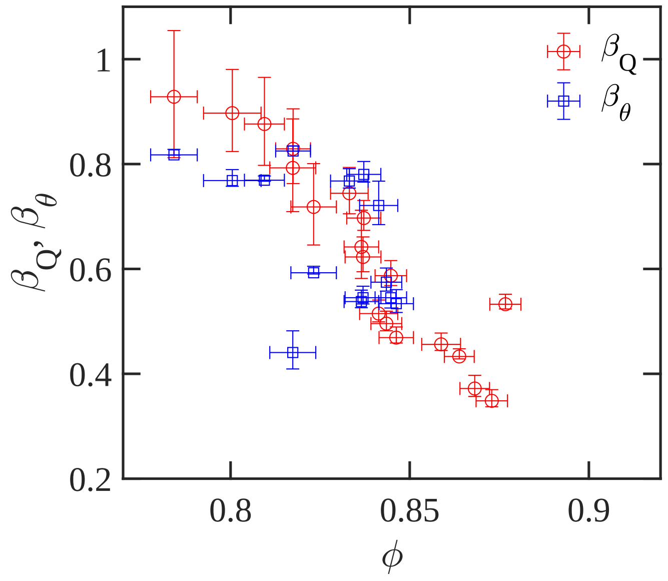

FIG. S5. Stretched exponential parameter β. The estimates of β_Q and β_θ from the stretched exponential fitting, $Q(\Delta t) = f_Q e^{-(\Delta t/\tau_Q)^{\beta_Q}} + a_Q$ and $C_\theta(\Delta t) = f_\theta e^{-(\Delta t/\tau_\theta)^{\beta_\theta}} + a_\theta$ (Fig. S4 and Fig. S7, respectively) are shown. The exponent values are closer to 1 for lower area fractions ϕ, indicating that the relaxation becomes closer to that of simple liquids.

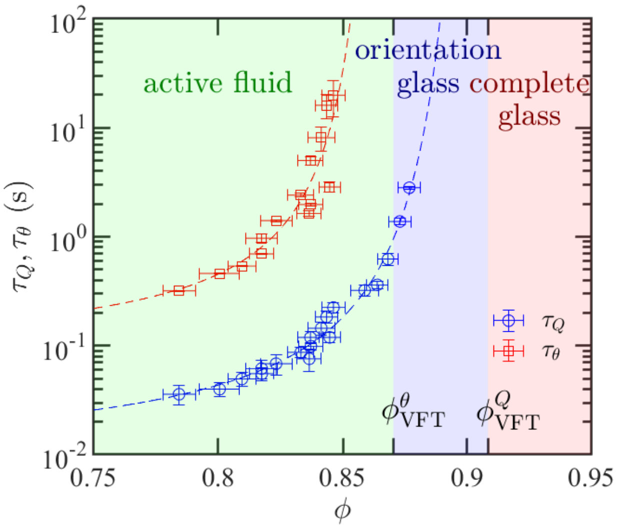

FIG. S6. Vogel-Fulcher-Tamman (VFT) fitting of the relaxation times. The data of τ_Q and τ_θ displayed in Fig. 2c are fitted here with the VFT law, $\tau \sim \exp\left(\frac{c\phi}{\phi_{\mathrm{VFT}}-\phi}\right)$ (dashed lines). The transition points are evaluated at $\phi^Q_{\mathrm{VFT}} = 0.908(12)$ for the translational relaxation and $\phi^\theta_{\mathrm{VFT}} = 0.870(35)$ for the orientational relaxation. The two-step transition scenario is also confirmed by the VFT fitting.

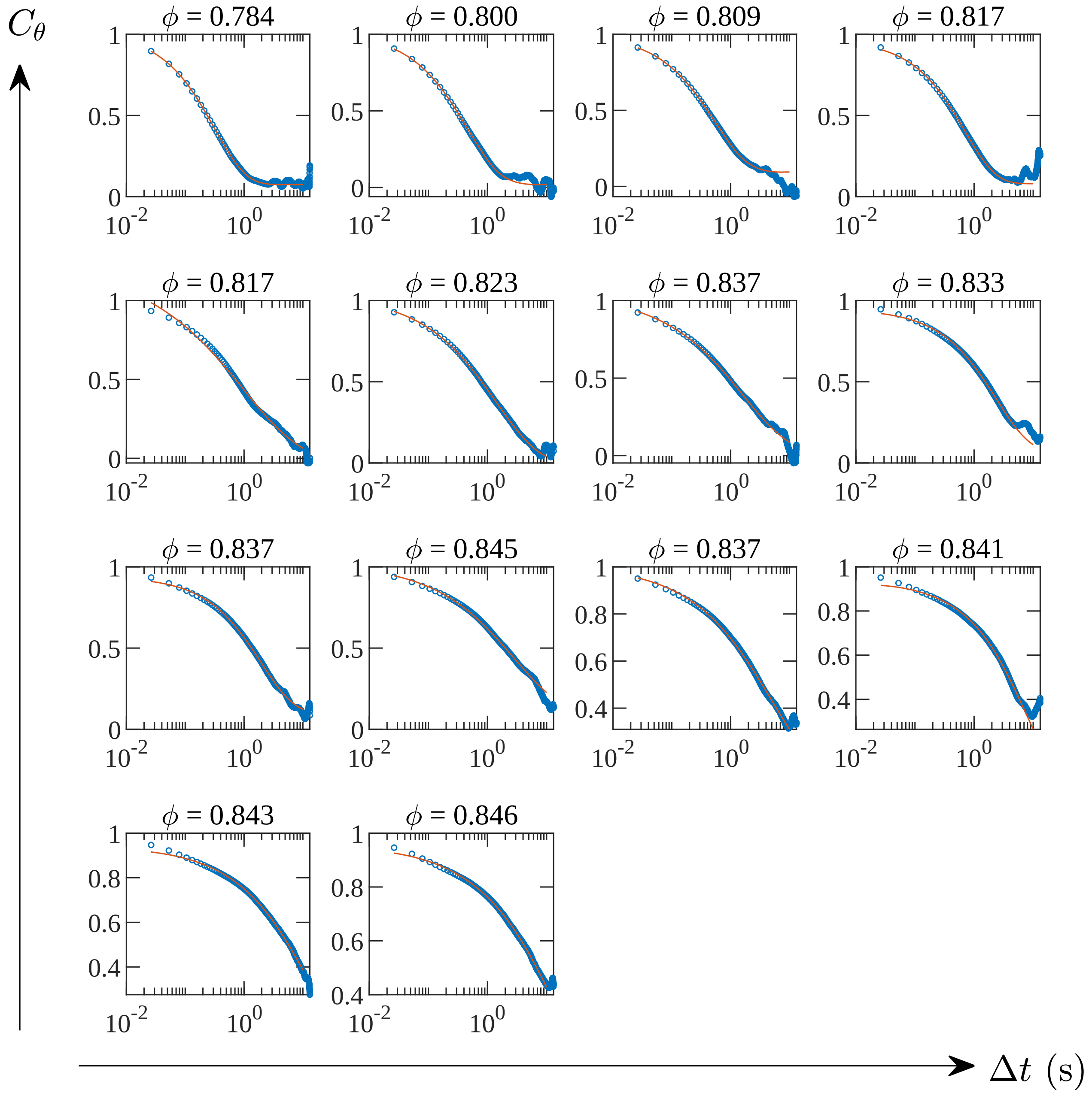

FIG. S7. Results of fitting of the orientational correlation function $C_\theta(\Delta t)$ by a stretched exponential function. Each data was fitted by a stretched exponential function plus an offset, $C_\theta(\Delta t) = f_\theta e^{-(\Delta t/\tau_\theta)^{\beta_\theta}} + a_\theta$. See Fig. S5 for the obtained values of β_θ.

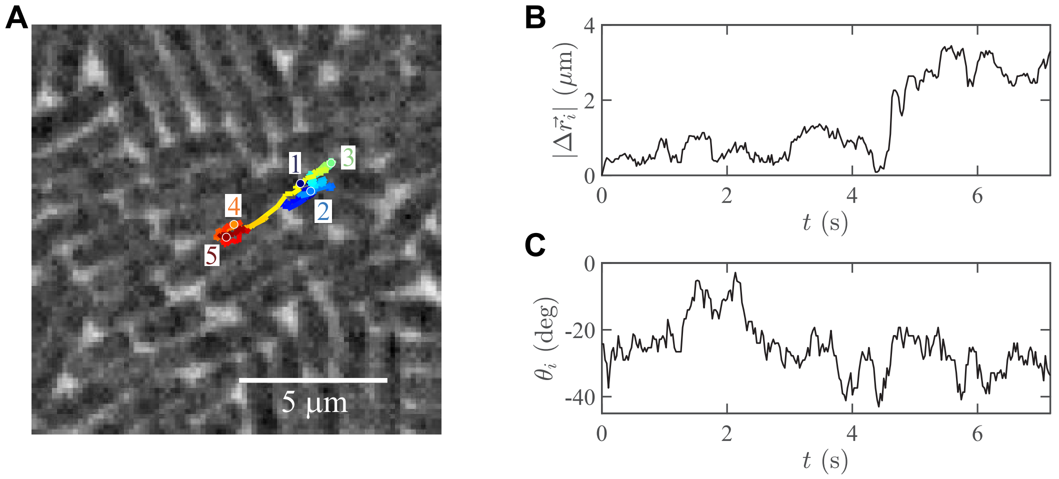

FIG. S8. A cage escape event in the orientation glass phase, $\phi = 0.873(4)$, different from the one shown in Fig. 3a-c. See also Movie 7. In this event, the cell did not move across a border of microdomains, unlike the event shown in Fig. 3a-c and Movie 6. A) Trajectory of the single cell for $0 \le t \le 7.16$ s, drawn on the phase-contrast image taken at the last time frame. Note that the origin of time is different from that used in Fig. 3a-c. The positions at $t = 0, 1.76, 3.53, 5.29, 7.06$ s are shown by colored disks with labels $1, 2, \cdots, 5$, respectively. B,C) Time series of the displacement from the initial position, $|\Delta\vec{r}_i(t)|$ (B), and that of the orientation $\theta_i(t)$ (C) of the cell tracked in panel A. These time series show a cage escape event during 4.5 s $\lesssim t \lesssim$ 4.7 s.

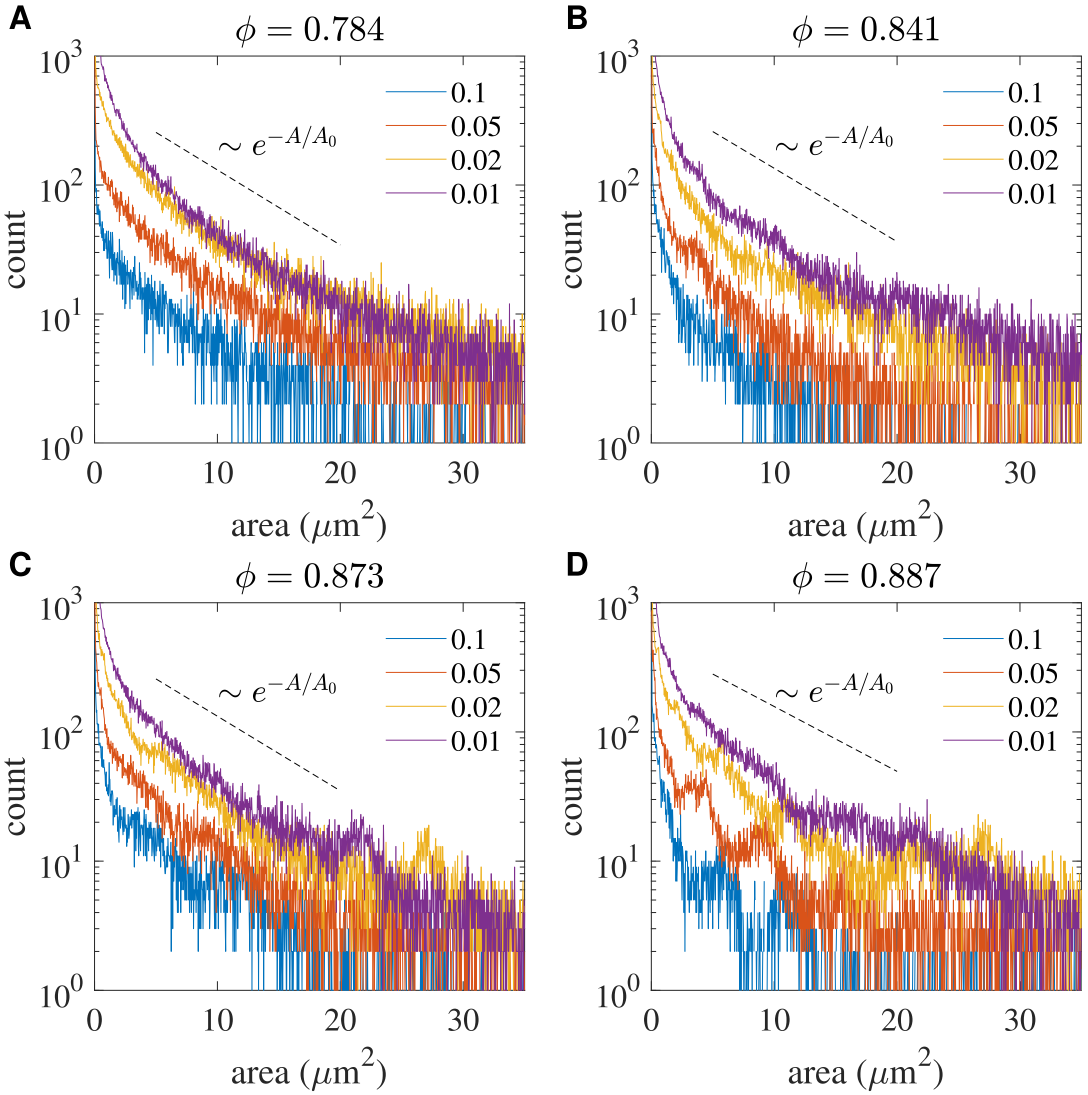

FIG. S9. Microdomain size distributions for different thresholds of $|\nabla\theta(\vec{r},t)|^2$ (legend, in the unit of $(\mathrm{rad}/\mu\mathrm{m})^2$) and for different area fractions ϕ. The dashed lines indicate the results of the fitting using all the displayed curves for each ϕ. The resulting values of the characteristic area A_0 are $A_0 = 7.5 \pm 1.8$ μm^2 (A), $A_0 = 7.6 \pm 2.6$ μm^2 (B), $A_0 = 7.5 \pm 2.5$ μm^2 (C), and $A_0 = 8.6 \pm 4.8$ μm^2 (D).

MOVIE CAPTIONS

Movie 1: Uninterrupted Movie of bacteria undergoing glass transitions. The Movie starts from the active fluid phase where bacteria were actively swarming, and lasts until they become completely jammed. The Movie shows a central region of size 259×214 pixels ($44.7\ \mu$m $\times$ $36.9\ \mu$m) and played at 20 times the real speed. Scale bar: 10 μm. Note that this Movie was taken from an experiment independent from the other observations, using a different substrate (well diameter 76.3(4) μm, depth $\approx 1.4\ \mu$m).

Movie 2: Movie of bacteria at $\phi = 0.784(7)$ (active fluid phase). Scale bar 5 μm. Played at real speed.

Movie 3: Movie of bacteria at $\phi = 0.841(5)$ (active fluid phase, close to ϕ_c^θ). Scale bar 5 μm. Played at real speed.

Movie 4: Movie of bacteria at $\phi = 0.873(4)$ (orientation glass). Scale bar 5 μm. Played at real speed.

Movie 5: Movie of bacteria at $\phi = 0.887(4)$ (complete glass). Scale bar 5 μm. Played at real speed.

Movie 6: The cage escape event in the orientation glass phase, $\phi = 0.873(4)$, shown in Fig. 3a-c. The left panel shows the trajectory of the single cell drawn on the phase-contrast image. The right panels show the time series of the displacement from the initial position, $|\Delta\vec{r}_i(t)|$ (top) and that of the orientation $\theta_i(t)$ (bottom) of the cell shown in the left panel. The cage escape event took place during 5 s $\lesssim t \lesssim$ 7 s.

Movie 7: Another cage escape event in the orientation glass phase, $\phi = 0.873(4)$, shown in Fig. S8. The left panel shows the trajectory of the single cell drawn on the phase-contrast image. The right panels show the time series of the displacement from the initial position, $|\Delta\vec{r}_i(t)|$ (top) and that of the orientation $\theta_i(t)$ (bottom) of the cell shown in the left panel. The cage escape event took place during 4.5 s $\lesssim t \lesssim$ 4.7 s.

Movie 8: Structure and evolution of microdomains at $\phi = 0.784(7)$ (active fluid phase). The left and right panels show the orientation field $\theta(\vec{r}, t)$ and its gradient squared $|\nabla\theta(\vec{r}, t)|^2$, respectively, overlaid on the phase-contrast image. Played at real speed. See also Fig. 4.

Movie 9: Structure and evolution of microdomains at $\phi = 0.841(5)$ (active fluid phase, close to ϕ_c^θ). The left and right panels show the orientation field $\theta(\vec{r}, t)$ and its gradient squared $|\nabla\theta(\vec{r}, t)|^2$, respectively, overlaid on the phase-contrast image. Played at real speed. See also Fig. 4.

Movie 10: Structure and evolution of microdomains at $\phi = 0.873(4)$ (orientation glass). The left and right panels show the orientation field $\theta(\vec{r}, t)$ and its gradient squared $|\nabla\theta(\vec{r}, t)|^2$, respectively, overlaid on the phase-contrast image. Played at real speed. See also Fig. 4.

Movie 11: Structure and evolution of microdomains at $\phi = 0.887(4)$ (complete glass). The left and right panels show the orientation field $\theta(\vec{r}, t)$ and its gradient squared $|\nabla\theta(\vec{r}, t)|^2$, respectively, overlaid on the phase-contrast image. Played at real speed. See also Fig. 4.
